# Optimal Surgical Strategies for Posterior Malleolar Ankle Fractures: A Morphology-Based Approach

**DOI:** 10.7759/cureus.81325

**Published:** 2025-03-28

**Authors:** Ting-Yu Chang, Chih-Wei Chang, Yen-Nien Chen

**Affiliations:** 1 Department of Orthopedics, Taipei Veterans General Hospital, Taipei, TWN; 2 Department of Orthopedics, Kuo General Hospital, Tainan, TWN; 3 Department of Orthopedics, National Cheng Kung University Hospital, Tainan, TWN; 4 Department of Physical Therapy, Asia University, Taichung, TWN

**Keywords:** clinical outcomes, fracture morphology, posterior malleolar fractures, radiographic review, surgical fixation, surgical strategies

## Abstract

Background

Surgical fixation of posterior malleolar fractures (PMFs) has traditionally been guided by fragment size. Recent studies have proposed fragment morphology as a more effective determinant for optimal fixation. To determine the optimal strategy for PMFs, we conducted a radiographic review of our patients.

Methods

Between January 2016 and December 2019, all adult patients with ankle fractures who underwent surgical fixation at our institution were reviewed (n=648). The exclusion criteria included pilon fractures, tumors, or infections causing neuromuscular dysfunction, insufficient follow-up, bilateral involvement, and prior injuries. After applying these criteria, 110 eligible patients remained for the study. They were further categorized on the basis of treatment type (non-fixation, screw, and plating) and fracture classification system (Haraguchi, Lauge-Hansen, and Weber classifications). The fracture fragment involvement was further classified as large (>25% of the articular surface) or small (<25%). The outcomes were evaluated via the modified Kellgren-Lawrence grade and the final malleolar step-off to assess osteoarthritis severity and fixation stability.

Results

Compared to non-fixation treatment, surgical intervention was associated with better outcomes in the Haraguchi type 1, supination-external rotation (SER), and pronation-external rotation (PER) groups. Additionally, surgical fixation using plates resulted in a lower step-off rate compared to screws. Notably, patients with small-fragment fractures who underwent surgical fixation exhibited outcomes comparable to or more favorable than those with larger fragments. These findings suggest that fracture morphology with size may have greater prognostic significance than fragment size alone.

Conclusion

This study on PMFs highlights that fracture morphology, rather than fragment size alone, should guide surgical decision-making for PMFs. Different PMF patterns call for personalized surgical strategies. Compared with screw fixation and non-fixation treatment, plate fixation yields superior outcomes, particularly in terms of joint space narrowing and stability. This study advocates for a morphology-based approach to managing PMFs, prioritizing plate fixation to achieve better results.

## Introduction

Ankle fractures account for approximately 9% of all fractures, with posterior malleolar fractures (PMFs) comprising 10% to 44% of these injuries. The most common mechanism is supination-external rotation (SER) [1,2]. In ankle fractures, untreated PMFs may lead to posttraumatic osteoarthritis, characterized by abnormalities such as joint step-offs, narrowing of the ankle joint space, and osteophyte formation [3].

Traditionally, surgical fixation of PMFs is recommended when the fragment involves more than 25% of the articular surface, as measured on lateral radiographs [3-5]. However, this size-based criterion may oversimplify the clinical decision-making process, and consequently, the optimal timing and methodology for PMF fixation remain controversial [6,7]. With respect to biomechanics, plating provides better stability for PMFs [8,9], and Bartoníček et al. [10] first proposed the use of a buttress plate to provide stable fixation. However, its clinical superiority over conventional screw fixation requires further investigation.

Haraguchi et al. classified rotational-type PMFs on the basis of CT images at the tibial plafond level, and three subtypes were recognized [11]. Studies suggest that morphology, rather than size, is an independent predictor of patient outcomes and should guide the choice of fixation method [12]. Notably, no current studies have conclusively identified the best surgical method for each morphological subtype.

The primary aim of this study was to refine the conventional criterion of >25% articular involvement by taking fracture morphology into treatment algorithms for PMFs. We hypothesized that morphological characteristics, obtained from either computed tomography (CT) or extrapolated from plain radiographs, would serve as a more ideal guide to appropriate fixation methodology.

## Materials and methods

Study design and period

With the approval of the Institutional Review Board, National Cheng Kung University Hospital, we conducted a retrospective study of patients with procedural codes 64272C and 64273C, which are specific to surgical fixation of multimalleolar fractures, between 2016 and 2019 at our hospital.

Inclusion and exclusion criteria

**Figure 1:**
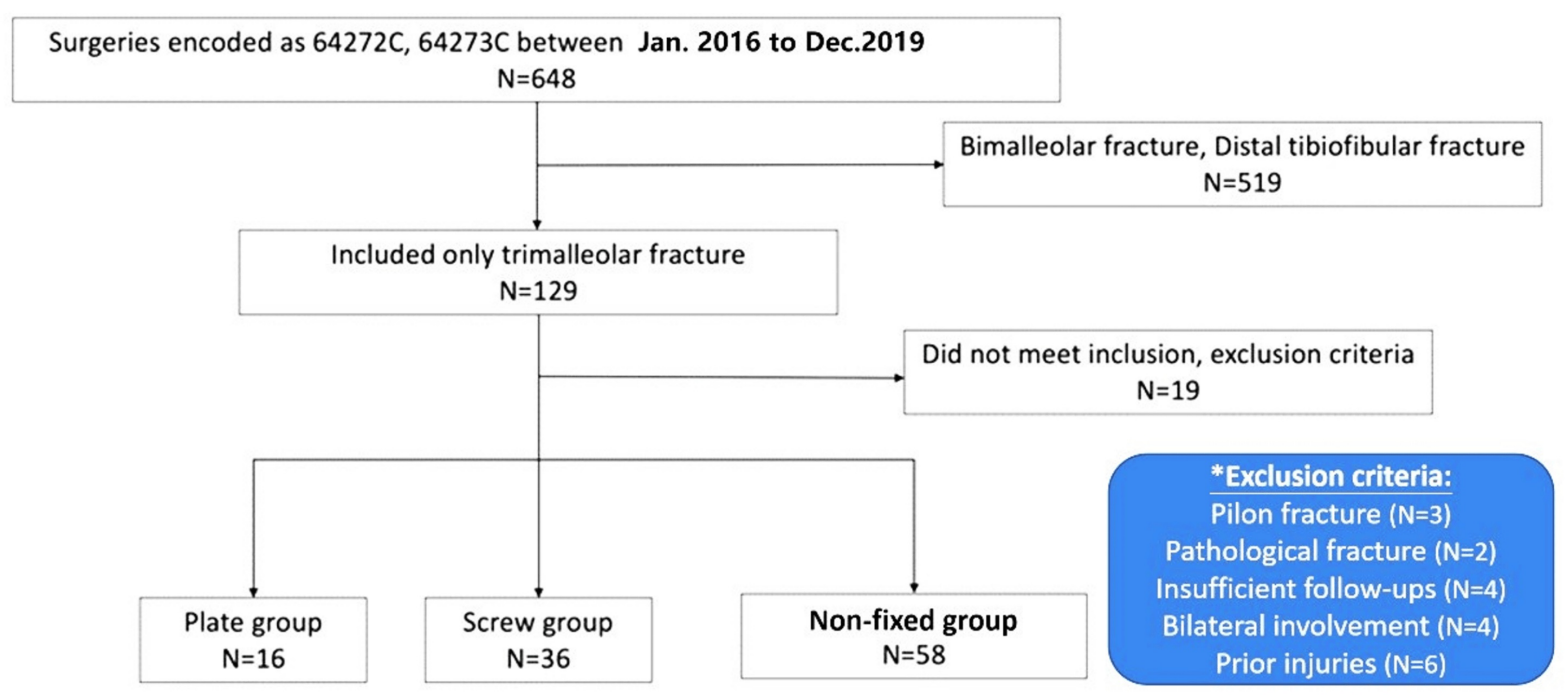
Flow diagram of eligible patients and exclusions, resulting in the study population

We screened medical records and radiographs to identify adult candidates suffering unilateral ankle fractures involving the posterior malleolus during the study period, as shown in Figure 1. The exclusion criteria were pilon fractures, clinical follow-up of less than six months, prior trauma to the ipsilateral lower limb, prior or concurrent neuromuscular dysfunction affecting the evaluation, and concurrent tumor or infection status.

Surgical interventions

All surgical fixation procedures were performed by 12 trained orthopedic surgeons experienced in trauma surgery. Patients received either posterior-anterior (PA) or anterior-posterior (AP) lag screws, plate fixation, or non-fixation treatment for the PMFs.

For the plate group, patients were positioned in either the prone or lateral decubitus position, depending on the surgeon’s preference and the location of the fracture fragment. Fixation was performed through either a posterolateral or posteromedial approach, selected to optimize visualization and access to the fracture site. Various plates were used for internal fixation of the displaced PMFs as a buttress, including the Synthes Variable Angle LCP Ankle Trauma System (Paoli, PA, USA), the Aplus Distal Fibula Double Hook Locking Plate System (Taipei, Taiwan), and the Hung Chun Plate System O103 (Taipei, Taiwan). 

In the screw group, the posterior malleolus was accessed through medial, lateral, or posterolateral incisions. Medial or lateral incisions were used for AP screw fixation. For lag screws, the anterior cortex was overdrilled to achieve the lag effect. The posterior fragment was identified by retracting the Achilles tendon medially and the peroneal tendons laterally. One to two lag screws were placed from posterior to anterior into the distal tibial metaphysis. The lateral or medial malleolus was repaired after the posterior fracture was fixed. Fragment positions and screws were confirmed via C-arm radiographs before wound closure in all patients.

For the non-fixation group, patients had trimalleolar fractures but only underwent fixation for the medial and/or lateral malleolus, leaving the PMF unfixed. The posterior malleolar fragment was allowed to heal through conservative management.

Radiographic evaluation

**Figure 2:**
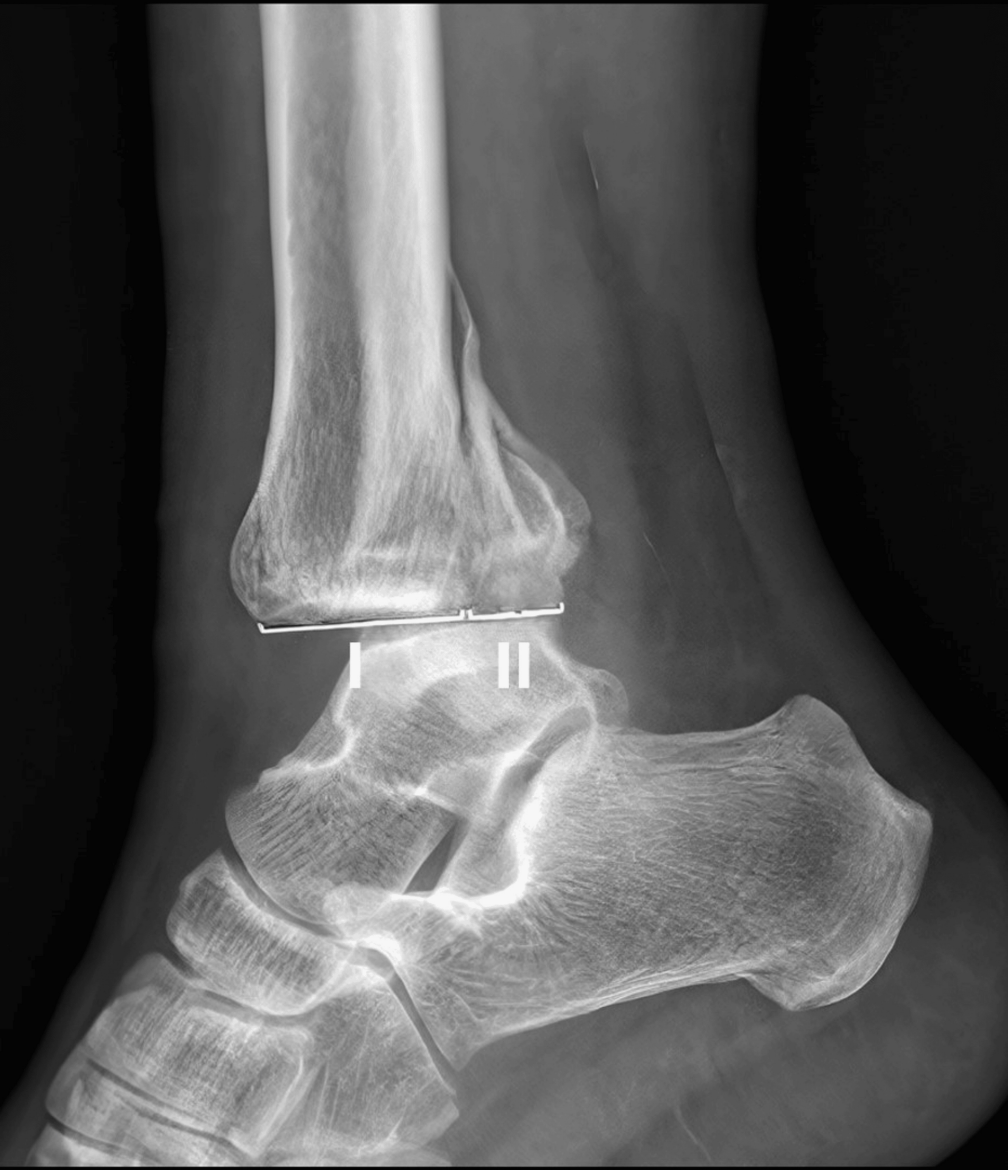
Measurement of fragment size via lateral view The percentage of the involved articular surface is calculated as II / (I + II). Based on the fracture fragment size and 25% joint surface involvement, patients were classified into large and small fracture groups.

We obtained lateral and mortise radiographs, both pre-surgery and post-surgery, for evaluation. For pre-operative evaluation, the traditional measurement method on lateral plain radiographs was used, as shown in Figure 2. Based on the size of the fracture fragment and 25% involvement of the joint surface, the patients were further classified into large fracture and small fracture groups. We assessed morphological characteristics using the Weber and Lauge-Hansen (L-H) classification systems, derived from high-quality lateral and mortise radiographic views, which have demonstrated reasonable correlation with CT in fracture characterization. In cases where CT imaging was available, fractures were further subclassified according to the Haraguchi classification into three types: type 1, characterized by a posterolateral-oblique fragment; type 2, involving medial extension into the posteromedial tibial plafond; and type 3, consisting of a small shell-type fragment limited to the posterior lip of the tibia.

The severity of postoperative arthritis was evaluated via the modified Kellgren-Lawrence (K-L) grade, as provided by Kraus et al. [13]. Standard anteroposterior and lateral radiographs of the affected ankle were reviewed by two independent observers. Joint space narrowing (JSN) was defined based on the visual reference atlas published by Kraus et al., without comparison to the contralateral side. K-L grading incorporated JSN, osteophytes, talar tilt, and subchondral sclerosis in a composite score (range 0-4). We also evaluated the step-off of the posterior malleolus as an outcome measure. Postoperative radiographs used for this evaluation were obtained at a minimum of six months post-surgery and no later than eight months for all included patients.

Statistical analysis

All the statistical analyses were performed via SPSS Statistics for Windows, Version 17 (Released 2008; SPSS Inc., Chicago, United States). Continuous variables are presented as the means ± standard deviations. The Kruskal-Wallis test was used to compare grading between groups. For intergroup comparisons of continuous variables that did not follow a normal distribution, the Mann-Whitney U test was utilized. The Pearson chi-square test was used to compare categorical variables between the groups. Statistical significance was set at p < 0.05.

## Results

During the study period, a total of 110 patients were included in this study. The patients' demographic characteristics are shown in Table 1. CT images were available for 62 patients and were classified according to the Haraguchi classification system; 44 patients had Haraguchi type 1 fractures, 11 patients had type 2 fractures, and 7 patients had type 3 fractures. Including those with CT images, all 110 patients were classified according to the L-H and Weber classification systems utilizing the radiographs. L-H SER and Weber B fractures accounted for the majority of fractures. Patients were further stratified based on fragment size and joint surface involvement, leading to the classification of 64 cases as large fractures and 46 as small fractures.

**Table 1:** Baseline demographics, fracture morphologies, and corresponding treatments SER: supination external rotation; PER: pronation external rotation; PA: pronation abduction; SAD: supination adduction * indicates statistical significance Analysis of patient data showed no significant differences among the groups in terms of gender, age, or length of hospital stay. However, smaller fractures were more prevalent in the conservative treatment group, whereas larger fractures were more commonly treated with plates. This indicates that our hospital's treatment approach aligns with traditional guidelines.

Characteristic	Plate	Screw	Non-fixation	Total	p-value
Gender					0.50
Male	6	8	16	30	
Female	10	28	42	80	
Age	50.4±17.6	50.4±14.9	49.8±13.2	50.1±14.4	0.98
Inpatient stay	3.9±1.4	5.4±3.9	4.7±4.5	4.7±3.4	0.18
Articular involvement %	35.9±9.6	33.4±9.1	23.1±11.3	28.3±10.3	0.00*
Weber classification					0.59
A	0	0	1	1	
B	10	26	45	81	
C	6	10	12	28	
Lauge-Hansen classification					0.13
SER	11	28	51	90	
PER	3	5	7	15	
PA	2	3	0	5	
SAD	0	0	0	0	
Haraguchi classification					0.07
Type 1	11	13	20	44	
Type 2	4	4	3	11	
Type 3	0	0	6	7	

Joint space narrowing and fixation methods

As shown in Table 2, for the most common fracture morphologies, L-H SER and Weber B plate fixation resulted in the least JSN on both lateral and AP views, showing a significant difference compared to other treatments. The most severe JSN was observed in patients who underwent non-fixation treatment, followed by those treated with screw fixation.

**Table 2:** Clinical outcomes versus various fracture morphologies K-L grade: Kellgren and Lawrence grade; JSN: joint space narrowing; AP: anterior-posterior; ROI: removal of implants; SER: supination external rotation; PER: pronation external rotation; PA: pronation abduction * indicates statistical significance There were no patients who underwent conservative treatment for Lauge-Hansen (L-H) posterior-anterior (PA) fractures, and no patients who received plate or screw fixation for Haraguchi type 3 fractures; therefore, data for these groups is not presented.

Fracture morphology	Management	n (%)	Age	Art. surface (%)	K-L grade						Step off n (%)	ROI n (%)	Re-surgery n (%)	Wound poor heal n (%)
					Total	Osteophyte	Lateral-JSN	AP-JSN	Talar tilt n (%)	Talar Scl n (%)				
Dannis Weber Classification														
B	Plate	12 (14.6)	51.8 (SD: 16.8)	38.5 (SD: 9.5)	1.33 (SD: 0.65)	1.33 (SD: 0.78)	0.83 (SD: 0.84)	0.50 (SD: 0.52)	0 (0)	0 (0)	4 (33.3)	4 (33.3)	5 (41.7)	3 (25)
	Screw	27 (32.9)	49.3 (SD: 15.8)	33.2 (SD: 9.7)	1.37 (SD: 0.69)	11.41 (SD: 0.75)	0.85 (SD: 0.53)	0.81 (SD: 0.48)	0 (0)	0 (0)	12 (44.4)	2 (7.4)	6 (22.2)	2 (7.4)
	Non-fixation	43 (52.4)	49.8 (SD: 12.2)	21.6 (SD: 6.8)	1.77 (SD: 0.95)	1.58 (SD: 0.70)	1.28 (SD: 0.59)	1.19 (SD: 0.55)	4.7 (2)	9.3 (4)	22 (51.2)	NA	19 (44.2)	5 (11.6)
	P		0.833	<0.000*	0.247	0.636	0.011*	<0.000*	0.395	0.149	0.533	0.06	0.165	0.297
C	Plate	6 (21.4)	47.5 (SD: 20.3)	33.8 (SD: 9.7)	1.83 (SD: 0.98)	1.67 (SD: 0.52)	1.17 (SD: 0.75)	1.00 (SD: 1.10)	0 (0)	33.3 (2)	1 (16.7)	2 (33.3)	2 (33.3)	2 (33.3)
	Screw	10 (35.7)	53.1 (SD: 12.6)	35.1 (SD: 7.5)	1.90 (SD: 1.10)	1.80 (SD: 0.79)	0.90 (SD: 0.99)	1.00 (SD: 1.25)	0 (0)	40 (4)	9 (90)	1 (10)	2 (20)	1 (10)
	Conservative	12 (42.9)	49.8 (SD: 17.0)	23.3 (SD: 8.3)	1.33 (SD: 0.65)	1.33 (SD: 0.78)	1.08 (SD: 0.29)	1.08 (SD: 0.29)	0 (0)	8.3 (1)	3 (25)	NA	5 (41.7)	1 (8.3)
	P		0.852	0.015*	0.467	0.311	0.468	0.543	NA	0.202	0.002*	0.304	0.555	0.321
Lauge-Hansen Classification														
SER	Plate	13 (14.4)	49.3 (SD: 18.5)	38.2 (SD: 9.2)	1.31 (SD: 0.63)	1.31 (SD: 0.75)	0.85 (SD: 0.80)	0.54 (SD: 0.52)	0 (0)	0 (0)	4 (30.8)	4 (30.8)	5 (38.5)	3 (23.1)
	Screw	29 (32.2)	49.8 (SD: 15.3)	33.1 (SD: 9.4)	1.48 (SD: 0.79)	1.48 (SD: 0.79)	0.90 (SD: 0.56)	0.83 (SD: 0.54)	0 (0)	3.4 (1)	14 (48.3)	2 (6.9)	6 (20.7)	3 (10.3)
	Non-fixation	48 (53.3)	48,7 (SD: 13.5)	21.3 (SD: 6.6)	1.67 (SD: 0.93)	1.50 (SD: 0.68)	1.27 (SD: 0.57)	1.17 (SD: 0.52)	2 (4.2)	5 (10.4)	23 (47.9)	NA	20 (41.7)	5 (10.4)
	P		0.902	<0.000*	0.563	0.858	0.017*	<0.000*	0.409	0.287	0.512	0.063	0.163	0.434
PER	Plate	3 (20)	54.7 (SD: 20.3)	31.4 (SD: 11.7)	2.33 (SD: 1.16)	1.67 (SD: 0.58)	1.33 (SD: 1.16)	1.00 (SD: 1.73)	0 (0)	2 (66.7)	1 (33.3)	1 (33.3)	1 (33.3)	2 (66.7)
	Screw	5 (33)	46.2 (SD: 13.7)	33.0 (SD: 7.3)	1.80 (SD: 1.30)	1.60 (SD: 0.89)	0.80 (SD: 1.30)	0.80 (SD: 1.30)	0 (0)	3 (60)	0 (0)	0 (0)	1 (20)	0 (0)
	Non-fixation	7 (46.7)	57.3 (SD: 8.9)	26.5 (SD: 9.4)	1.71 (SD: 0.76)	1.71 (SD: 0.95)	1.00 (SD: 0.00)	1.14 (SD: 0.38)	0 (0)	0 (0)	2 (28.6)	0 (0)	4 (57.1)	1 (14.3)
	P		0.29	0.76	0.65	0.95	0.43	0.39	NA	0.037*	0.037*	0.375	0.418	0.065
PA	Plate	2 (40)	51.0 (SD: 15.6)	37.3 (SD: 12.2)	1.50 (SD: 0.70)	0.20 (SD: 0.00)	1.00 (SD: 0.00)	1.00 (SD: 0.00)	0 (0)	0 (0)	0 (0)	1 (50)	1 (50)	0 (0)
	Screw	3 (60)	62.3 (SD: 9.0)	41.2 (SD: 7.0)	1.33 (SD: 0.58)	1.67 (SD: 0.58)	0.67 (SD: 0.58)	1.33 (SD: 1.53)	0 (0)	0 (0)	2 (66.7)	1 (33.3)	1 (33.3)	0 (0)
	P		0.25	0.56	0.74	0.41	0.41	1	NA	NA	0.3	0.7	0.7	NA
Haraguchi Classification														
Type 1	Plate	13 (29.5)	52.2 (SD: 16.6)	37.0 (SD: 10.6)	1.38 (SD: 0.77)	1.38 (SD: 0.77)	1.00 (SD: 0.82)	0.54 (SD: 0.52)	0 (0)	1 (7.7)	2 (15.4)	3 (23.1)	4 (30.8)	3 (23.1)
	Screw	14 (31.8)	53.7 (SD: 14.1)	34.2 (SD: 10.2)	1.29 (SD: 0.91)	1.21 (SD: 0.80)	0.71 (SD: 0.61)	0.79 (SD: 0.89)	0 (0)	2 (14.3)	7 (50)	1 (7.1)	2 (14.3)	2 (14.3)
	Non-fixation	17 (38.6)	53.4 (SD: 11.4)	22.4 (SD: 6.9)	2.00 (SD: 1.00)	1.59 (SD: 0.62)	1.35 (SD: 0.70)	1.35 (SD: 0.70)	1 (5.9)	3 (17.6)	8 (47.1)	NA	6 (35.3)	3 (17.6)
	P		0.95	0.033*	0.08	0.34	0.07	0.01*	0.44	0.73	0.12	0.27	0.4	0.84
Type 2	Plate	4 (36.4)	52.3 (SD: 17.4)	37.4 (SD: 8.1)	2.00 (SD: 0.82)	1.75 (SD: 0.50)	0.75 (SD: 0.96)	1.00 (SD: 1.41)	0 (0)	1 (25)	3 (75)	3 (75)	3 (75)	2 (50%)
	Screw	4 (36.4)	58.5 (SD: 11.2)	30.5 (SD: 21.4)	1.25 (SD: 0.50)	1.25 (SD: 0.50)	1.00 (SD: 0.00)	1.00 (SD: 0.00)	0 (0)	0 (0)	3 (75)	0 (0)	2 (50)	0 (0)
	Non-fixation	3 (27.3)	24.1 (SD: 6.7)	48.7 (SD: 9.6)	2.00 (SD: 0.00)	2.00 (SD: 1.00)	1.00 (SD: 0.00)	1.00 (SD: 0.00)	0 (0)	0 (0)	2 (66.7)	0 (0)	2 (66.7)	0 (0)
	P		0.58	0.15	0.16	0.33	0.7	0.7	NA	0.38	0.96	0.07	0.76	0.12
Type 3	Non-fixation	6 (100)	41.2 (SD: 13.0)	16.5 (SD: 49.7)	1.17 (SD: 0.41)	1.17 (SD: 0.41)	1.17 (SD: 0.41)	1.00 (SD: 0.00)	0 (0)	0 (0)	2 (33.3)	NA	4 (66.7)	1 (16.7)
Fragment Size														
Large	Plate	16 (25)	50.8 (SD: 18.5)	38.7 (SD: 8.6)	1.44 (SD: 0.73)	1.44 (SD: 0.73)	0.87 (SD: 0.81)	0.69 (SD: 0.79)	0 (0)	1 (6.3)	5 (31.3)	6 (37.5)	7 (43.8)	5 (31.3)
	Screw	32 (50)	52.3 (SD: 13.9)	35.9 (SD: 7.5)	1.47 (SD: 0.84)	1.47 (SD: 0.76)	0.84 (SD: 0.68)	0.88 (SD: 0.79)	0 (0)	4 (12.5)	19 (59.4)	2 (6.3)	7 (21.9)	3 (9.4)
	Non-fixation	16 (25)	51.4 (SD: 14.5)	30.7 (SD: 5.3)	1.87 (SD: 1.20)	1.81 (SD: 0.91)	1.38 (SD: 0.72)	1.25 (SD: 0.68)	2 (12.5)	2 (12.5)	5 (31.3)	NA	6 (37.5)	2 (12.5)
	P		0.96	0.01*	0.51	0.35	0.06	0.04*	0.05	0.79	0.08	0.01*	0.25	0.13
Small	Plate	2 (4.3)	47.0 (SD: 8.5)	23.0 (SD: NA)	2.00 (SD: 1.41)	1.50 (SD: 0.71)	1.50 (SD: 0.71)	0.50 (SD: 0.71)	0 (0)	1 (50)	2 (100)	0 (0)	2 (100)	2 (100)
	Screw	5 (10.9)	37.6 (SD: 16.6)	20.0 (SD: 6.1)	1.80 (SD: 0.84)	1.80 (SD: 0.84)	1.00 (SD: 0.71)	0.80 (SD: 0.45)	0 (0)	0 (0)	2 (40)	1 (20)	1 (20)	0 (0)
	Non-fixation	39 (84.8)	49.2 (SD: 12.8)	18.4 (SD: 4.0)	1.59 (SD: 0.75)	1.41 (SD: 0.60)	1.18 (SD: 0.45)	1.13 (SD: 0.41)	0 (0)	3 (7.7)	20 (51.3)	NA	18 (46.2)	4 (10.3)
	P		0.29	0.23	0.71	0.48	0.41	0.02*	NA	0.09	0.34	0.71	0.26	0.67

When accounting for fragment size, despite having larger fragments, the plate fixation group still had the least JSN, whereas conservative treatment led to the most severe narrowing even with smaller posterior malleolar fragments.

Outcomes based on Haraguchi classifications

For Haraguchi type 1 patients, those posterior malleolar fragments that were left non-fixed had a smaller percentage of articular involvement (p=0.033) but ended up with significantly more severe JSN on AP view (p=0.001) as detailed in Table 2. There were no significant differences between treatments for Haraguchi type 2, and as all Haraguchi type 3 fractures were managed non-operatively, no interventional comparison was performed for this group.

Patients with Weber C fractures and L-H pronation-external rotation (PER) fractures who underwent plate fixation had significantly lower step-off rates compared to those treated with screws or non-fixation management (p=0.002, p=0.037). Notably, among patients with Weber C fractures, 9 out of 10 who underwent screw fixation experienced step-off deformities at follow-up, whereas only one out of six patients in the plate fixation group exhibited step-off.

Others

Regarding the need for secondary surgery, no significant differences were observed among the three treatment groups across different fracture morphologies. However, in cases involving large fracture areas, plate fixation was associated with a higher likelihood of requiring a second procedure for implant removal compared to screw fixation (p=0.01).

## Discussion

To determine how different fractures respond to various treatments for PMFs, the authors conducted a retrospective image analysis of various fracture morphologies. Our findings indicated that plate fixation provided the best treatment outcomes among the three fracture types: Lauge-Hansen classification SER, Weber B, and Haraguchi type 1. Additionally, although there were no significant differences in outcomes for patients with PER or PA morphology within the L-H classification, a trend suggested that plate fixation still resulted in better outcomes than other treatment methods did.

**Figure 3:**
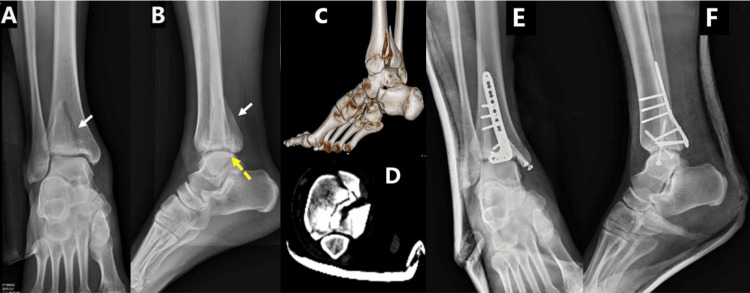
Surgical fixation of a Haraguchi type 2 posterior malleolar fracture using a plate A 58-year-old male sustained a fall from a height of 2 meters, resulting in a right ankle fracture involving the posterior malleolus. Preoperative radiographs (A and B) showed the fracture, with white arrows indicating the affected area. Although the lateral view (B, yellow dotted arrow) suggested minimal involvement of the articular surface, a CT scan (C, D) revealed medial extension of the fracture, classifying it as Haraguchi type 2. To address this, a posteromedial approach with plate fixation was chosen for internal fixation. Postoperative imaging confirmed the successful restoration of the articular surface (F). A, B: Preoperative AP and lateral radiographic views; C: 3D reconstruction based on a CT scan; D: Transverse section of the CT scan at the plafond level; E, F: Postoperative AP and lateral views

In 2020, Blom et al. noted that clinical outcomes for PMF type 2 patients are generally worse than those for patients with type 1 or type 3 [12]. Therefore, it was assumed that PMF type 2 would benefit the most from plate fixation (Figure 3). However, our results suggest that plate fixation is associated with better outcomes only for Haraguchi type 1 fractures. Although previous studies have suggested that PMF type 2 fractures benefit the most from plate fixation, our findings indicate that the fractures most likely to benefit from plate fixation are Haraguchi type 1, L-H SER, and Weber B, as these were the most common fracture patterns within their respective classifications. While Haraguchi type 2 fractures did not show a statistically significant advantage, a trend favoring plate fixation was observed in this study. This may be attributed to the limited sample size. Based on the study's primary outcomes, we recommend plate fixation for all fracture types, except for fractures like Haraguchi type 3, which represents a small shell morphology and may not derive the same level of benefit.

In this study, large PMF fragments were more likely to receive surgical intervention, adhering to traditional guidelines for fixing PMFs when they involve more than 25% of the tibial plafond. However, both large and small fragment groups demonstrated similar outcomes across the different treatments, with no significant differences between them. This finding underscores that fragment size alone is insufficient for determining the optimal treatment strategy. While a >25% articular surface involvement on lateral radiographs is traditionally considered the threshold for fixation, our results suggest that morphology provides more clinically relevant insights. For example, although both may be classified as small fragments, a Haraguchi type 2 fragment that extends medially - commonly seen in PER fractures - has greater biomechanical significance than a thin posterior shell typically observed in type 3 fractures. Therefore, even when CT is unavailable, morphology assessed using standardized lateral and mortise radiographs may better guide the choice between plate fixation, screw fixation, or non-fixation compared to relying solely on fragment size [14].

In addition to morphology, fracture fragment displacement is critical in determining the need for surgical fixation in PMFs. Traditionally, a displacement threshold of greater than 2 mm on lateral radiographs has been used as a key indication for fixation, along with fragment size exceeding 25-30% of the tibial plafond. However, defining an absolute cut-off for displacement in clinical imaging remains challenging, particularly in cases with complex fracture morphology. Standard lateral radiographs provide only a two-dimensional evaluation, which may not accurately reflect the true extent of displacement in axial or coronal planes. This issue is even more pronounced in three-dimensional imaging, where a reliable method for quantifying displacement is lacking. Future studies should focus on developing standardized displacement measurement protocols and incorporating three-dimensional assessment techniques to enhance surgical decision-making for PMFs.

Our results support the use of plates in certain patients. Although patients who undergo plate fixation generally have larger posterior fragments, this method provides better stability and outcomes, including lower step-off rates and reduced JSN, than screws do, particularly in Haraguchi type 1, L-H SER, and Weber B fractures. The advantages of plate fixation stem from multiple factors. Karaca et al. [15] highlighted the importance of anatomical reduction and rigid internal fixation for the posterior malleolus to achieve satisfactory clinical and functional outcomes. Proper reduction of the articular surface is crucial for preventing post-traumatic arthritis.

Studies have shown that both screws and plates can achieve adequate reduction [16,17]. Non-fixation treatment, however, is not recommended for a majority of PMF subtypes [10]. Xing et al. [18] analyzed 30 patients with dislocated trimalleolar fractures and complex PMFs, finding satisfactory outcomes after three months. Patients treated with plate fixation had better Short Musculoskeletal Function Assessment Questionnaire (SMFA) discomfort scores and showed trends toward improved functional and mobility subscores [19].

However, plates have certain drawbacks. In addition to being more expensive, plates placed close to the distal tibia may protrude at the most distal part, causing irritation to surrounding soft tissues, such as the flexor hallucis longus. Unlike screws, plates cannot sink the head, which may lead to discomfort. Our study also revealed a higher rate of implant removal requests among patients with PMFs fixed with plates.

Limitations

Several limitations warrant consideration in the interpretation of our findings. First, the insufficient sample size of patients with specific fracture morphologies prevented their inclusion in the analysis. Additionally, procedural heterogeneity arose due to variations in surgical techniques among the 12 participating surgeons, and the plates used were not specifically designed for posterior malleolar fixation. Although individual follow-up durations were not presented in a dedicated table, all patients underwent clinical evaluation for a minimum of six months postoperatively, with the majority followed for approximately one year. Radiographic assessments, focusing on early degenerative changes, were conducted within a standardized early postoperative window, rather than evaluating long-term functional outcomes. Future studies incorporating prospective mid- to long-term follow-ups, advanced imaging techniques, and anatomically designed plates are warranted to provide stronger evidence in clinical practice.

## Conclusions

This retrospective radiographic assessment highlights the significance of fracture morphology, rather than fragment size, in guiding surgical strategies for PMFs. Plate fixation provides superior joint stability and outcomes compared to screws or non-fixation but is associated with a higher removal rate. Personalized approaches based on fracture patterns can enhance patient care. Future research should prioritize advanced imaging, mid-to-long-term follow-ups, and anatomically designed plates to strengthen clinical evidence.
